# Emotional and Cognitive Empathy in First-Year Medical Students

**DOI:** 10.1155/2013/801530

**Published:** 2013-10-21

**Authors:** Sandra Dehning, Sarah Gasperi, Daniela Krause, Sebastian Meyer, Eva Reiß, Max Burger, Fabian Jacobs, Anna Buchheim, Norbert Müller, Matthias Siebeck

**Affiliations:** ^1^Department of Psychiatry and Psychotherapy, Ludwig Maximilian University, Nußbaumstraße 7, 80336 Munich, Germany; ^2^Department of Surgery, Ludwig Maximilian University, Nußbaumstraße 20, 80336 Munich, Germany; ^3^Institute of Psychology, University of Innsbruck, Innrain 52, 6020 Innsbruck, Austria

## Abstract

*Objectives*. Doctors' empathy towards their patients is considered important for treatment outcome. However, during medical school there might be a decline in empathy called “hardening of the heart.” This study evaluated the cognitive and emotional empathy in medical students and investigated the influence of a preference for a specialty and students attachment styles. *Methods*. 126 first-year medical students were included and completed the Reading the Mind in the Eyes Test revised version (RME-R), the Balanced Emotional Empathy Scale (BEES), and the Experiences in Close Relationships-Revised Adult Attachment Questionnaire (ECR-R). *Results*. Students identified 22 ± 4.30 of 36 photographs in the RME-R test correctly (norm: 26). The female students' mean BEES total score was 51.1 ± 17.1 and the male students' 27.2 ± 22.6; *P* < 0.0001. The female students' mean BEES score was significantly (*P* = 0.0037) below the female norm of 60. Students who preferred a specialty with continuity of patient care scored significantly higher in the BEES (*P* = 0.014). A more avoidant attachment style was associated with a lower BEES score (*P* = 0.021). *Conclusion*. The students showed low emotional and cognitive empathy scores and an avoidant attachment style. This supports the inclusion of specific training in cognitive and emotional empathy in medical education.

## 1. Introduction

Patients usually speak without interruption for less than 90 seconds when they sit down to talk to their doctor, but the way the doctor behaves during this initial period sets the tone for trust and disclosure in the doctor-patient relationship [1]. Doctors' empathy towards their patients is considered important not only to ensure that they are good listeners, but also to facilitate delivering bad news or assisting their patients in coping with their illness. Research has shown that medical students have an average or above-average level of empathy at the beginning of medical school. However, the phenomenon of “hardening of the heart” [2], with a decline in empathy during the course of medical school, has been reported in several studies [3–5]. It has therefore been suggested that medical education should include approaches to retain and enhance empathy [6]. 

 The phenomenon of empathy, which includes the ability to understand the mental and affective states of others, has been described as a cornerstone of our lives as “social animals” [7]. There is a growing body of research on empathy in medical school (for an overview see [8]); however, this research has mostly focused on the cognitive aspect of empathy in terms of perspective-taking and “standing in the patient's shoes.” In social cognitive neuroscience, the term “cognitive empathy” is frequently used synonymously with “cognitive perspective-taking” or “Theory of Mind.” 

To date, research on empathy in medical students has used self-administered paper-and-pencil tests with Likert scales. Such an approach is associated with suggestible and socially expected responses and therefore may not deliver reliable results. In the present study we used an established Theory of Mind task, the “Reading the Mind in the Eyes” Test (RME-R Test) [9], to evaluate cognitive empathy. The RME-R Test carries no risk of suggestibility and is a widely used instrument in studies on the neurobiological substrates of social and emotional skills [10]. In addition to evaluating cognitive empathy, we examined emotional empathy with the Balanced Emotional Empathy Scale (BEES) [11], a scale that has previously been used in medical students [2].

 The objectives of our study were to evaluate whether first-year medical students show high levels of cognitive and emotional empathy, whether a preference for a specialty continuous patient care predicts a high empathy score, and whether students' attachment style is related to empathy levels. 

## 2. Materials and Methods

### 2.1. Setting/Participants

The study sample consisted of 126 first-year medical students at the Ludwig Maximilian University, Munich, Germany. The data were collected after a compulsory lecture (anatomy) to ensure that as many of the first-year students as possible were present. The principal investigators explained the study procedures and remained with the participants while they completed the questionnaires, which took about 20 minutes. The ethics committee of the Ludwig Maximilian University, Munich, approved the study. The participants were not required to give informed consent to participate in the study.

### 2.2. Measures

#### 2.2.1. The Reading the Mind in the Eyes Test

The Reading the Mind in the Eyes Test revised version (RME-R Test) [9] is an established Theory of Mind task that assesses cognitive empathy. The test consists of 36 photographs depicting only the eye region of Caucasian individuals. A rectangular area of approximately 5 × 2 inches delineates the eye region, encompassing the entire width of the face from midway up the nose to right above the brow. Names of four complex emotional states (one target word and three foils) are presented at each corner of the photograph. The task is to attribute an emotional state to the person in the picture on the basis of expressions in the eye region alone. Because the test was administered in English, a detailed glossary was appended to the test that explained all the adjectives by using synonyms and example sentences. 

#### 2.2.2. Balanced Emotional Empathy Scale

The 30-item Balanced Emotional Empathy Scale (BEES) [11] is a reliable and valid instrument consisting of 30 positively or negatively worded items (15 items in each category) that measure responses to fictional situations and particular life events (e.g., “I cannot feel much sorrow for those who are responsible for their own misery”). The test evaluates the extent to which the respondent can feel the suffering of others or take pleasure in their happiness. Participants rate their agreement or disagreement with each of the 30 items on a 9-point Likert scale ranging from −4 to 4 (resulting in a total score ranging from −120 to 120). Higher scores represent higher levels of emotional empathy.

#### 2.2.3. Experiences in Close Relationships-Revised (ECR-R) Adult Attachment Questionnaire

We used the Experiences in Close Relationships-Revised (ECR-R) Adult Attachment Questionnaire (13) to examine whether adult attachment orientations are associated with empathy scores. The questionnaire is a 36-item self-report measure of adult attachment style comprising an 18-item attachment-related anxiety scale (e.g., “I worry about being abandoned”) and an 18-item attachment-related avoidance scale (e.g., “I prefer not to show a partner how I feel deep down”). Each item is rated on a 7-point Likert scale. Students rated the extent to which each item described their experiences in close relationships from “not at all” (1) to “very much” (7). Higher scale values, obtained as the mean of the respective 18 items, indicate greater anxiety or avoidance, respectively.

#### 2.2.4. Personal Characteristics

We designed a questionnaire to record personal characteristics that included questions on gender, age, family background, number of close relationships (people with whom they feel comfortable about discussing very personal matters), active membership in a religious community, involvement in a social network like Facebook, and interest in one of two types of medical specialty (specialty with continuity of patient care, e.g., family medicine, paediatrics, internal medicine, etc., or specialty with less interpersonal contact, e.g., surgery, radiology, pathology, etc.). 

### 2.3. Analysis

In addition to the usual descriptive statistics (reported as mean ± standard deviation unless otherwise stated), we evaluated the relationship between relevant sociodemographic factors and scores in the BEES and RME-R Test with Welch's *t*-tests or two-way ANOVA *F*-tests (type II), adjusting for gender. We also calculated Pearson or Spearman correlations as appropriate. One-sample *t*-tests were used to compare the score distributions in our student sample with published norms. Significance was defined as *P* < 0.05. We used linear regression models and regression trees [12] to identify sociodemographic characteristics associated with empathy as measured by the BEES and RME-R Test. In the linear regression models, we used backward stepwise selection procedures based on Akaike's Information Criterion [13] to trade off between model complexity and goodness-of-fit. Here, the age of the students was categorized as 18-19, 20–24, and >24, since this improved the model fitness compared to a linear age effect. We checked the validity of the regression models by inspecting the residuals of the fitted models (normal Q-Q plot) and assessing multicollinearity by the variance inflation factor; see [14], for example. In the other inferential approach, the study population was recursively partitioned into subsets resulting from binary splits, each according to the input variable with the strongest association with the response variable. This hierarchical approach enabled the identification of interactions between variables and specific effects in subgroups of students. In this analysis, associations were evaluated with permutation tests and a univariate significance level of 5%, where splits were only allowed when the resulting subsets both contained at least 10 students.

 We defined the minimum number of items that had to be answered in order for a questionnaire to be included as follows: 33% of the subscale-specific items for the BEES and the ECR-R and 50% of the items in the RME-R Test. None of the questionnaires had to be excluded (104 students had complete data for the BEES and the ECR-R and the remaining questionnaires had only one or two missing items). Missing values in the BEES were resolved by imputing the mean of the observed items for that particular participant; this was done separately for the positively and negatively worded items. Similarly, missing values in the ECR-R were imputed by the mean of the available items of the corresponding subscale. In the RME-R test, missing answers were interpreted as “the participant did not recognize the emotion.” Because of the small number of imputations, we did not conduct a sensitivity analysis with only complete cases.

 All analyses were performed with the statistical software environment R 2.11.1 [15]. 

## 3. Results

All questionnaires were completed according to the respective instructions [9, 11, 16]. The sample consisted of 126 first-year medical students, 36 (29%) women and 90 (71%) men. The mean age was 21 years. For further sample description, see Table 1. 

### 3.1. The Reading the Mind in the Eyes Test

The students identified 22 ± 4.30 (8–32) of the emotional states in the 36 photographs correctly. The male students identified 21.6 ± 4.34 correctly and the female students 23.1 ± 4.04; the number of correct answers did not differ significantly between males and females (*P* = 0.07). In Baron-Cohen's study (9), the score for the males in the normal adults sample was 26.0 ± 4.2 and for the females 26.4 ± 3.2.

**Figure 1:**
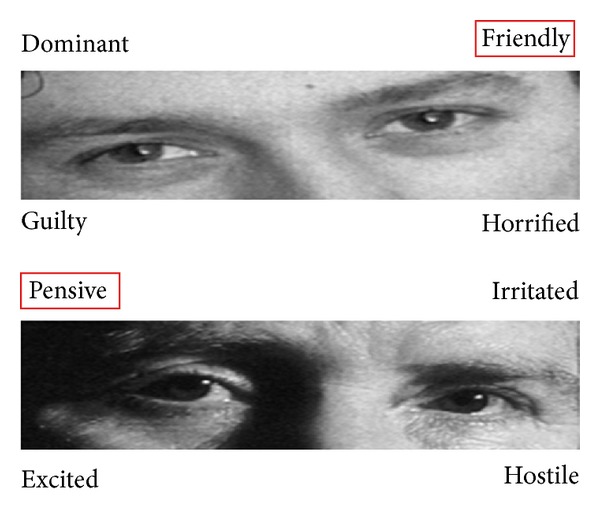
Emotions correctly identified most and least often by the first-year medical students.

 In our sample, the emotion “friendly” was most often identified correctly (by 92.1% of the students; see Figure 1) and the emotion “defiant” most often incorrectly (by 29.4% of the students). Male students wrongly identified the emotion “pensive” most frequently (25.6%) (see Figure 1) and female students the emotion “doubtful” (25.0%). 

 There was a weak positive correlation between performance in the RME-R Test and the number of close relationships (Spearman's *ρ* = 0.19, *P* = 0.035). Specifically, students with up to two close relationships recognized a mean of 18.1 (male) and 19.3 (female) emotions, whereas students with more than two close relationships recognized a mean of 22.3 (male) and 23.4 (female) emotions (*P* < 0.0001, *F* test). This characteristic was also the strongest predictor in the regression model (*P* < 0.0001) for the RME-R Test (see Table 2).

### 3.2. The Balanced Emotional Empathy Scale

The female students had a mean BEES total score of 51.1 ± 17.1 (range: 10–82), whereas the male students scored 27.2 ± 22.6 (range: −30–90). It is noteworthy that the mean BEES score of the female students in our sample was significantly (*P* = 0.0037) lower than the female norm of 60 (11), whereas the male students' mean score was comparable to the male norm of 29 (*P* = 0.46).

In line with the results for the RME-R Test, a greater number of close relationships was associated with higher emotional empathy scores. Specifically, mean BEES scores for the male students were 15.6 (≤2 close relationships) versus 29.7 (>2 close relationships) and for the female students 36.7 versus 52.4 (*P* = 0.0066, *F* test).

**Figure 2:**
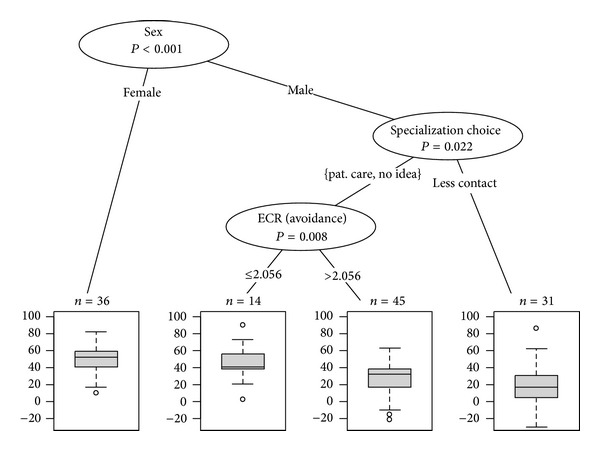
Regression tree for the BEES developed with sociodemographic data and the ECR-R subscales as input variables. The terminal nodes show the number of students for the respective subgroup, together with a boxplot of the BEES.

The analysis also showed a significant association between the intended specialization and empathy scores among male students: those who preferred a specialty with continuity of patient care scored significantly higher in the BEES than those who preferred a specialty with less interpersonal contact (*P* = 0.014). There was no evidence for such an association (*P* = 0.29) among the female students. According to the regression tree (Figure 2), gender was the most important classification factor for the BEES, as described above. The subsample of male students who preferred a specialisation with continuity of patient care or who had not decided on a specialization could be further discriminated by the score on the ECR-R avoidance subscale, with an optimal cut point at 2.056, indicating that a more avoidant attachment style is associated with lower emotional empathy levels (*P* = 0.008). This result is supported by the negative correlation between the ECR-R avoidance subscale and the BEES among all male students (*r* = −0.24; *P* = 0.021). Although the linear regression model (Table 3) indicated a trend towards more emotional empathy with an increasing score on the ECR-R anxiety subscale, there was no significant relationship between these scales (*P* = 0.07).

More than two close relationships and age below 20 were also significant predictors of higher BEES scores among the students, which is in agreement with the results of the RME-R Test analysis. 

We found a moderately positive correlation between the BEES scores and performance in the RME-R Test. This correlation was statistically significant for both males (*r* = 0.35, *P* = 0.0007) and females (*r* = 0.39, *P* = 0.018).

### 3.3. Experiences in Close Relationships-Revised (ECR-R) Adult Attachment Questionnaire

The mean values were 3.14 ± 1.10 for avoidance (norm 2.93) and 3.29 ± 1.06 for anxiety (norm 3.64). We found no significant differences between male and female students. The correlation between the two subscales was *r* = 0.56 (*P* < 0.0001). 

## 4. Discussion

To the best of our knowledge this study was the first to investigate cognitive and emotional empathy and the attachment style of first-year medical students. On the basis of previous studies we expected to find above-average empathy scores. However, the students showed comparatively low scores in emotional and cognitive empathy (measured with the BEES and RME-R Test, resp.) and, in comparison with the stated norms of the ECR-R, an avoidant (*P* = 0.038) attachment style. 

 Our study subjects scored lower in the advanced adult Theory of Mind RME-R Test (22 out of 36 eye expressions identified correctly) than the sample described by Baron-Cohen et al. [9]. The mean age of the general population controls in their study was 46.5, which at first sight might suggest that cognitive empathy may increase with age. However, the sample also included students studying for undergraduate degrees at Cambridge University, with a mean age of 20.8; these students scored even higher (28.0 out of 36) than the control group of “normal adults.” 

 The students in our sample with more than two close relationships scored higher on the empathy tests than those with two or fewer close relationships. This might suggest that attributing a mental state to a person is learned through interpersonal contact. 

 We found a significant correlation between the results of the BEES and the RME-R Test: students with higher BEES scores recognized more items correctly. This finding may indicate an association between emotional and cognitive empathy. 

 The BEES scores in our sample were lower than the norms, possibly indicating critical or cautious response behaviour. In a study on emotional empathy in first-year medical students from the South Central United States [2] that also used the BEES, male first-year medical students had empathy scores significantly above the norm, regardless of their specialty choice (male total score 37.87 ± 22.42). A possible explanation for the significantly higher scores in the South-Central United States sample might be the smaller number of students per year, leading to a less competitive and more friendly atmosphere. Similar to the United States students, the Munich students showed a significant association between the preferred specialty and the BEES: students preferring a specialisation with continuity of patient care scored higher than those interested in a specialisation with less interpersonal contact (*P* = 0.014). Attachment-avoidant students in the ECR-R who had less than two close relationships had lower BEES scores. This finding is in accordance with studies that found a negative correlation between attachment avoidance and empathy [17–19]. Wei et al. [20] suggested an explanation for this negative correlation: they argued that subjects with high levels of attachment avoidance may have diminished knowledge of the inner experience of others because of their tendency to avoid connecting with others. 

The study could be limited by the fact that neither the BEES nor the RME-R were designed specifically for medical students. Furthermore, the BEES included socially desirable answers. The use of English language versions of the tests may have made them somewhat more difficult for the students to complete, even though synonyms and explanatory sentences were provided. Fortunately, however, the nonverbal and intuitive structure of the RME-R Test reduced suggestibility and decreased the risk of participants giving socially desirable responses.

What is the relevance of the finding that only 41% of the students declared an interest in a specialisation with continuity of patient care? The examples for a specialty with continuity of patient care were “family medicine, paediatrics, internal medicine, and so forth.” and for a specialty with less interpersonal contact, “surgery, radiology, pathology, and so forth.” Thus, this finding might suggest that the students' motivation for studying medicine was the idea of a (financially) secure and prestigious job or a greater interest in technical skills rather than a purely altruistic or patient-oriented decision. Having an empathetic mind is not a precondition for becoming a medical student. Teaching empathetic behaviour to medical students could help ensure that all doctors gain the necessary patient-related skills. 

 In recent years, there has been a shift in the focus of medical education in Germany such that the medical curriculum is increasingly focusing on teaching basic patient interaction skills. The first step was a new medical licensing act in 2002, which was implemented in the winter term of 2003/2004 [21]. One aim of the new act was to focus more on the practical relevance of patient communication. Additionally, the curriculum includes more small-group-orientated didactics; this could encourage more emotional empathy and cognitive perspective-taking. These changes in the curriculum may help improve German medical students' social skills and their ability to empathize. Our data supports the notion that medical schools should include cognitive perspective-taking and the development of empathizing skills for medical students.

 In future studies it would be interesting to compare international cohorts of medical students who are taught according to different medical curriculums.

## 5. Conclusion

In this study, the medical students showed low emotional and cognitive empathy scores and an avoidant attachment style. As empathy is a key competence for the doctor-patient relationship, the presented findings emphasize the importance of specific training in cognitive and emotional empathy in medical education.

## Figures and Tables

**Table 1:** Sociodemographic characteristics of the sample.

Gender	
Male	90 (71%)
Female	36 (29%)
Age	
Mean ± SD	21.0 ± 2.94
Range	18–33
18-19 yrs	39 (31%)
20–24 yrs	69 (55%)
>24 yrs	18 (14%)
Grown up with	
Mother	10 (8%)
Father	2 (2%)
Both	113 (90%)
Other	1 (1%)
Number of siblings (*n* = 124)	
Median (min–max)	1 (0–10)
Place of living (*n* = 124)	
At home with family	31 (25%)
Moved out	93 (75%)
Religious community	
Active member	40 (32%)
Nonactive member	86 (68%)
Number of close relationships	
1	2 (2%)
2	17 (13%)
3	19 (15%)
4	21 (17%)
5 or more	67 (53%)
Social networking with Facebook	
Yes	114 (90%)
No	12 (10%)
Daily internet time	
Less than 1 hour	36 (29%)
More than 1 hour	90 (71%)
Specialization choice (*n* = 125)	
Continuity of patient contact	51 (41%)
Less interpersonal contact	40 (32%)
No idea	34 (27%)
Own psychiatric or psychotherapeutic experience	
Yes	10 (8%)
No	116 (92%)

**Table 2:** Selected linear regression model for the RME-R test based on 124 students with complete data (*R* ^2^ = 0.21).

	Estimate	Std. error	*t* value	*P* value
(Intercept)	22.140	1.670	13.257	<0.0001
ECR (avoidance)	−0.462	0.329	−1.403	0.163
Age group 20–24	−0.084	0.801	−0.105	0.917
Age group >24	−2.525	1.155	−2.186	0.031
Number of close relationships >2	4.357	1.035	4.211	<0.0001
Having siblings	−2.253	1.122	−2.008	0.047

**Table 3:** Selected linear regression model for the BEES based on 125 students with complete data (*R* ^2^ = 0.38).

	Estimate	Std. error	*t* value	*P* value
(Intercept)	28.215	8.351	3.378	0.0010
ECR (avoidance)	−5.339	1.918	−2.784	0.0063
ECR (anxiety)	3.590	1.989	1.805	0.0736
Female gender	17.682	3.956	4.470	<0.0001
Specialization choice: patient care	4.608	4.321	1.066	0.2885
Specialization choice: less contact	−6.683	4.478	−1.492	0.1383
Age group 20–24	−11.280	3.983	−2.832	0.0055
Age group >24	−7.229	5.632	−1.284	0.2018
Number of close relationships >2	15.900	4.933	3.223	0.0017
